# Advantages and Limitations of ChatGPT in Healthcare: A Scoping Review

**DOI:** 10.1002/hsr2.71219

**Published:** 2025-09-11

**Authors:** Seyyede Fateme Ghasemi, Parastoo Amiri, Zahra Galavi

**Affiliations:** ^1^ Medical Informatics Research Center, Institute for Futures Studies in Health Kerman University of Medical Sciences Kerman Iran; ^2^ Department of Health Information Technology, School of Allied Medical Sciences Lorestan University of Medical Sciences Khorramabad Iran; ^3^ USERN Office Lorestan University of Medical Sciences Khorramabad Iran; ^4^ Department of Health Information Technology, School of Allied Medical Sciences Zabol University of Medical Sciences Zabol Iran

**Keywords:** advantages, ChatGPT, health, limitations, scoping review

## Abstract

**Background and Aims:**

ChatGPT, an AI language model developed by OpenAI, has emerged as a transformative tool in the healthcare sector. Its ability to process vast amounts of medical data and generate human‐like responses offers significant potential for enhancing patient engagement and supporting healthcare professionals. However, despite its advantages, there are significant limitations. This scoping review aims to critically evaluate both the advantages and limitations associated with integrating ChatGPT into healthcare.

**Methods:**

In this scoping review, we searched for the original articles published in online databases (PubMed, Web of Science, Scopus, and Google Scholar) from January 1, 2020, to May 30, 2023 using relevant keywords (“ChatGPT,” “Health,” “Advantage,” and “Limitation”) in English language. Data collection was done using a researcher's checklist using Excel version 2019 software.

**Results:**

Out of 4982 articles found, 28 articles were included in the study. All articles were conducted in 2023. The type of study in more than half of the articles was cross‐sectional. Advantages and limitations extracted from the studies were grouped into eight and nine categories, respectively. The most common advantages were “Clinical Decision Support (CDS)” and “improved medical education,” which included enhanced teaching methods and curriculum development. Specific benefits in medical education were comprehensive drug counseling, understanding of radiation oncology, and predicting drug‐drug interactions. Research assistance benefits were also notable, such as facilitating data analysis, selecting strong research ideas, and aiding in academic writing. The most frequent limitations were “knowledge limitations and accuracy” and “reliability.”

**Conclusion:**

This scoping review has provided a comprehensive analysis of the advantages and limitations of ChatGPT in the healthcare sector. While the model shows significant promise in enhancing CDS and improving medical education, it is essential to remain vigilant about its limitations, particularly concerning knowledge accuracy and reliability.

## 1 Introduction

The Chat Generative Pre‐Trained Transformer (ChatGPT) is an artificial intelligence (AI) based platform that was built and developed by OpenAI. This platform revolutionized the way that people interact with technology by using national language processing (NLP) and machine learning (ML) algorithms [1]. The platform attracted 100 million users only 2 months after its release [2]. ChatGPT is more advanced than previous chatbots or voice assistants, enabling users to engage in conversations with machines [3]. A large database makes this technology allow for personalized interactions, so making conversations with ChatGPT, feel like talking to a real person [4, 5]. Due to the high efficiency of ChatGPT, it is utilized in various fields, including healthcare [3].

One of the most challenging fields that ChatGPT has entered is healthcare. ChatGPT can have a deep influence on this field and it can contribute significantly to users' health [1, 6]. It can facilitate more natural conversations with patients by providing realistic responses. It can significantly affect various health fields such as teleconsultation [7], medical diagnosis [8], and drug prescription [9]. In the field of disease diagnosis, ChatGPT can analyze patient data and test results, assist doctors in diagnosing conditions, and provide treatment recommendations. It can also offer significant support in areas such as medical education and telemedicine. This technology can serve as a powerful tool in carrying out processes like managing medical documents, which can be time‐consuming and tedious but are highly important, thereby enhancing the quality and efficiency of these documents [10].

ChatGPT has demonstrated tremendous potential in the field of medicine, particularly in the areas of health education and health research [11]. A review of articles on ChatGPT in this domain suggests that it can bring numerous benefits. These benefits include improving the quality of scientific texts, enhancing healthcare‐related activities such as workflow optimization, cost savings, documentation, personalized medicine, and improving health literacy, as well as enhancing the quality of healthcare education [12].

Despite its numerous applications in healthcare, this technology requires access to precise and up‐to‐date medical data to respond accurately to users' questions. However, accessing current medical data conflicts with the principle of confidentiality, this problem is one of the challenging reasons for the acceptance of ChatGPT in healthcare [1, 13]. Furthermore, after collecting data from millions of users worldwide, this information can inadvertently be disclosed to other audiences and used for various purposes [14].

Another challenge for ChatGPT in healthcare is that its main structure is not designed to answer medical questions. Also, it doesn't have enough expertise and knowledge to understand the relationships between patient conditions and various treatments [15]. Garg et al. [8] represent that ChatGPT can be a suitable clinical assistance but it cannot be considered a reliable source of information and also it cannot replace the human brain. Nowrozy [16] in his study, pointed out the concerns of individuals following the emergence of AI in the context of human jobs. Borji [17] also highlighted significant issues regarding the use of ChatGPT, including that its output lacks accuracy in terms of scientific facts. The information it provides may seem credible, making it challenging for an informed specialist to detect inaccuracies, but it ultimately proves to be incorrect. Other concerns included issues related to privacy, ethical implications, and social consequences.

Many systematic reviews have been done on the challenges of ChatGPT in the healthcare [8, 12, 18, 19, 20]. These studies were limited in terms of the keywords and databases that searched [12]. Additionally, they considered only limited aspects of health, such as research [18]. Like any other technology, ChatGPT has its challenges, and physicians and healthcare organizations should be aware of its problems and limitations in this area. Understanding the benefits and disadvantages of this technology will lead to more informed use. It has not been completely identified how reliable ChatGPT can be in healthcare, but it is evident that ChatGPT is poised to revolutionize the lives of all people worldwide. In this scoping review article, we aim to review the challenges and limitations of ChatGPT in healthcare and evaluate its advantages and disadvantages.

## 2 Materials and Methods

The report for this scoping review article was prepared following the PRISMA‐ScR (Preferred Reporting Items for Systematic Reviews and Meta‐Analyzes extension for Scoping Reviews) guidelines [21]. Also, the reporting of results was done in accordance with Assel et al. recommendations to ensure data quality and transparency [22].

### 2.1 Eligibility Criteria

To ensure the selection of pertinent studies and to minimize bias, we defined specific inclusion and exclusion criteria. The inclusion criteria included: (1) studies conducted in 2023; (2) studies written in English; (3) studies that mentioned the advantages of using ChatGPT; and (4) studies that mentioned disadvantages of using ChatGPT.

In this study, the exclusion criteria were as follows: (1) studies categorized as “systematic reviews,” “editorials,” or “protocols”; (2) studies which the full text was not available; (3) studies written using ChatGPT; (4) studies conducted on nonhuman populations; and (5) studies involving chatbots other than ChatGPT. To ensure the chosen studies were relevant to the main research question and to reduce potential bias, the research team set specific inclusion and exclusion criteria. These criteria were used when reviewing the titles, abstracts, and full texts of articles sourced from databases. As a result, studies that met the inclusion criteria were included in the review, while others were excluded. This method allowed the research team to accurately select studies pertinent to the research topic and minimize bias in the results.

### 2.2 Information Sources and Search Strategy

First, the keywords required for developing the search strategy were selected based on a review of studies and the use of the Medical Subject Headings (MeSH). Specific strategies were formulated for each database (Supporting information File 1). This study used a range of databases, such as PubMed, Web of Science, Scopus, and Google Scholar. The search period was set from January 1, 2020, to May 30, 2023. Two groups of keywords were used to create the search strategy: the initial category comprised keywords associated with ChatGPT, while the second category focused on keywords pertinent to the healthcare sector. The primary terms utilized in this study were “ChatGPT,” “Health,” “Advantage,” and “Limitation.”

### 2.3 Selection of Sources of Evidence

To select the desired articles and remove those that involve the features of the exclusion criteria, all the extracted articles are first imported into the Mendeley software version 2.80.0. Duplicate articles were easily removed using Mendeley. Ryyan's website was used to review the remaining articles. Rayyan is an internet‐based platform that is suitable for article review and easy to use. The articles were uploaded on this site and their titles and abstracts were quickly screened. Two authors (ZG and FGh) independently reviewed the articles based on the eligibility criteria. The articles that remained unclear even after the abstract review were examined by the third author (PA). The full texts of the included articles were reviewed by two authors (ZG and FGh). All of these processes were conducted independently by them.

### 2.4 Data Items

To extract data from the remaining studies, we initially created a form using Microsoft Excel software 2019 version. This form included the following items for data extraction: article title, authors' names, publication year, study type, clinical area, study purpose, advantages, and limitations. Data extraction was done by two authors (ZG and FGh). After the initial two authors extracted data from the selected articles, all three authors met to reconcile findings and address any differences in their assessments. Any disagreements were resolved through a consensus‐based approach, which involved thorough discussions about the study. In summary, the review strategy for this scoping review was conducted using a collaborative framework involving all three reviewers. This methodology facilitated a thorough, objective, and rigorous scoping review, enhancing the robustness and reliability of the conclusions derived from the evidence.

## 3 Results

### 3.1 Selection of Sources of Evidence

**Figure 1:**
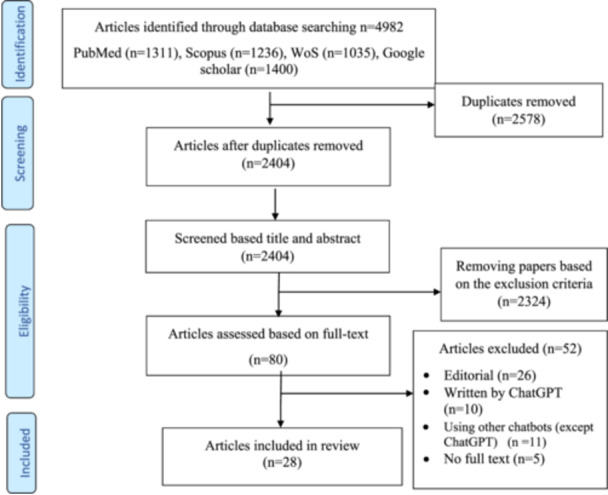
Flow diagram of study selection.

Initially, 4982 studies were identified. After duplicates were removed, 2404 studies remained and were screened based on their titles and abstracts. Of these, 80 studies underwent a full‐text review. Ultimately, 28 studies met our inclusion criteria, as shown in Figure 1.

### 3.2 Characteristics of Sources of Evidence

The characteristics of the studies are outlined in Table 1. Fifty percent of the studies focused on assessing ChatGPT's accuracy and reliability in responding to clinical questions from both patients and healthcare professionals [23, 24, 27, 28, 29, 31, 32, 36, 38, 39, 42]. Additionally, five studies identified the potential applications of ChatGPT in medical education and health science research [25, 33, 35, 43, 44]. Although the studies were not focused on a specific clinical field, the majority of them were conducted in the field of medical research. All studies were conducted in 2023, following the emergence of ChatGPT technology. More than half of the studies were cross‐sectional.

**Table 1:** Most significant characteristics of the included studies.

Author, year (reference)	Objective (s)	Type of study	Area of clinical medicine
Cao et al. 2023 [23]	Evaluating the accuracy of information provided by ChatGPT regarding liver cancer screening, surveillance, and diagnosis.	Retrospective study	Liver cancer
Xie et al. 2023 [24]	Evaluating the ability of ChatGPT to provide informative and accurate responses to a set of hypothetical questions designed to simulate an initial consultation about rhinoplasty.	Observational study	Rhinoplasty
Hosseini et al. 2023 [25]	Exploring perspectives on the interest and use of ChatGPT across education, research, and healthcare purposes.	Survey	Healthcare
Al‐Worafi et al. 2023 [26]	Identifying potential applications, benefits, and risks of using ChatGPT in medical and health sciences research.	Experimental study	Medical and health sciences research
Johnson et al. [27]	Evaluating the accuracy and comprehensiveness of ChatGPT‐generated responses to medical queries developed by physicians.	Cross‐sectional study	Medical information
Samaan et al. 2023 [28]	Examining ChatGPT accuracy and reproducibility in answering patient questions regarding bariatric surgery.	Cross‐sectional study	Bariatric surgery
Liu et al. 2023 [29]	Determining whether ChatGPT can generate useful suggestions for improving clinical decision support (CDS) logic and assessing non‐inferiority compared to human‐generated suggestions	Cross‐sectional study	Clinical decision support
Huang et al. 2023 [30]	Benchmarking the performance of ChatGPT on the American College of Radiation (ACR) radiation oncology in‐training (TXIT) exam and the Red Journal gray zone cases.	Benchmarking study	Radiation oncology
Branum & Schiavenato 2023 [31]	Assessing the reliability and accuracy of ChatGPT in providing answers to a complex clinical question.	Cross‐sectional study	Medical questions
Ali et al. 2023 [32]	Reporting the performance of the large language model ChatGPT in the context of lacrimal drainage disorders.	Cross‐sectional study	Lacrimal drainage disorders
Sallam et al. 2023 [33]	Investigating the pros and cons of ChatGPT use in medical, dental, pharmacy, and public health education.	Descriptive study	Medical, dental, pharmacy, and public health education
Sallam et al. 2023 [34]	Describing the output of ChatGPT regarding COVID‐19 vaccine conspiracy beliefs and compulsory vaccination.	Descriptive study	COVID‐19 vaccine
Haq et al. 2023 [35]	Contributing to the editorial principles on the possible use of AI‐based tools for scientific writing.	Exploratory study	Writing for health journals
Ayers et al. 2023 [36]	Evaluating the ability of ChatGPT to provide quality and empathetic responses to patient questions.	Cross‐sectional study	Patient questions about health
Balas & Ing 2023 [37]	Evaluating the use of ChatGPT for the diagnosis of ophthalmic disease and compare it to existing tools.	Cross‐sectional study	Ophthalmic disease
Uz & Umay 2023 [38]	Evaluating whether ChatGPT can be used to obtain information about common rheumatic diseases.	Cross‐sectional study	Rheumatic diseases
Zuccon & Koopman 2023 [39]	Investigating the impact of knowledge provided in the prompt versus the knowledge encoded in the model on ChatGPT responses.	Cross‐sectional study	
Almazyad et al. 2023 [40]	Investigating the efficacy of using ChatGPT‐4 in panel discussions as an AI chatbot to facilitate knowledge sharing and improve decision‐making.	Cross‐sectional study	Pediatric Palliative Care
Rao et al. 2023 [41]	Evaluating ChatGPT's capacity for clinical decision support in radiology via the identification of appropriate imaging services for breast cancer screening and breast pain.	Cross‐sectional study	Breast cancer
Ghosh & Bir 2023 [42]	Evaluating ChatGPT's aptitude for responding to higher‐order questions on medical biochemistry.	Cross‐sectional study	Medical biochemistry
Cascella et al. 2023 [15]	Highlighting the potential applications and limits of a large language model in healthcare.	Cross‐sectional study	Health (general)
Huang et al. 2023 [43]	Evaluating the performance of ChatGPT in key areas of clinical pharmacy practice, including prescription review, patient medication education, adverse drug reaction recognition, ADR causality assessment, and drug counseling.	Cross‐sectional study	Clinical pharmacy
Amri and Hisan 2023 [44]	Exploring the potential benefits of ChatGPT and Dall‐E in medical education and provide practical examples of their implementation.	Cross‐sectional study	Medical education
Juhi et al. 2023 [45]	Investigating the effectiveness of ChatGPT in predicting and explaining common drug‐drug interactions.	Cross‐sectional study	Drug‐drug interactions
Thirunavukarasu et al. 2023 [46]	Evaluating the strengths and weaknesses of ChatGPT in primary care using the Membership of the Royal College of General Practitioners Applied Knowledge Test (AKT) as a medium.	Observational study	Primary care
Shahsavar & Choudhury 2023 [6]	Investigating the factors influencing users' perception of decision‐making processes and intentions to use ChatGPT for self‐diagnosis and to explore the implications of these findings for the safe and effective integration of AI chatbots in health care.	Cross‐sectional survey	Self‐diagnosis
Liu et al. 2023 [47]	Determining if ChatGPT can generate useful suggestions for improving clinical decision support logic and to assess non‐inferiority compared to human‐generated suggestions.	Cross‐sectional study	Clinical decision support
Bulck and Moons 2023 [48]	Evaluating the trustworthiness, value, and danger of ChatGPT‐generated responses to virtual patient questions.	Survey	Patient questions about health

### 3.3 Advantages of ChatGPT in Healthcare

**Figure 2:**
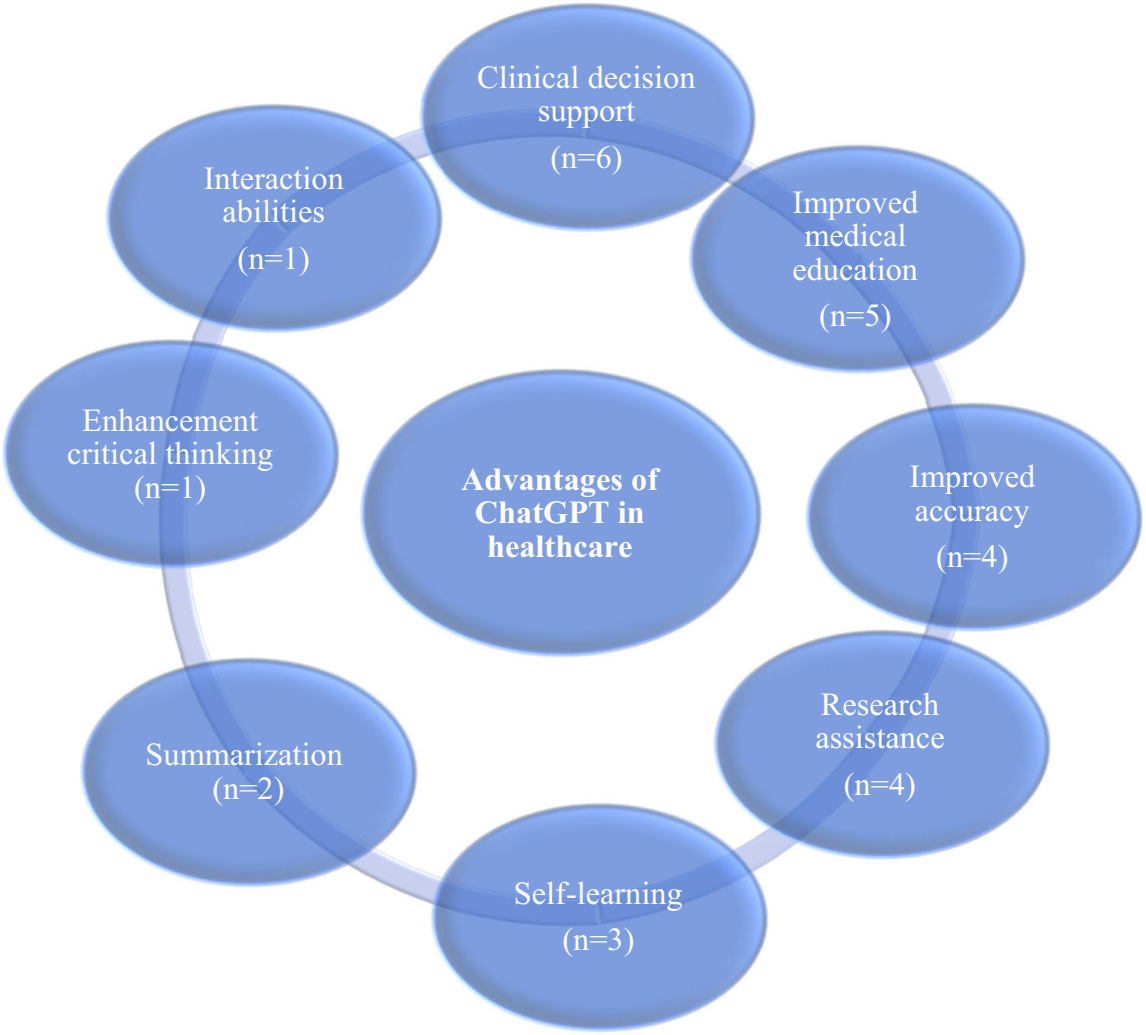
Advantages of ChatGPT in healthcare.

In Figure 2, the advantages of ChatGPT in healthcare are presented, and subsequently, the explanations for each advantage are stated.

- **Clinical decision support (CDS)**: In several included studies, ChatGPT demonstrated significant potential in improving CDS systems through its ability to learn from prior interactions and effectively utilize large language models. According to Liu et al. [29, 47], AI‐generated suggestions offered unique and relevant insights, although their overall utility and acceptance varied. These suggestions could play a vital role in optimizing CDS alerts, identifying potential improvements, and aiding experts in refining alert logic. This highlights ChatGPT's potential to enhance CDS logic and other areas requiring complex clinical decision‐making. Balas and Ing [37] emphasized that ChatGPT's ability to learn from past cases significantly improved diagnostic accuracy. The model adeptly handled various queries and provided real‐time provisional and differential diagnoses for common ophthalmic conditions. This capability supports primary care and emergency physicians in quickly evaluating, treating, and triaging patients. Uz and Umay [38] confirmed ChatGPT's reliability as a resource for information on common rheumatic diseases. Their study showed that the scales developed to measure the information's reliability and usefulness were consistent and dependable. Additionally, ChatGPT demonstrated an impressive ability to rectify errors and provide accurate guidance in clinical practice and public health discussions. Cascella et al. [15] highlighted ChatGPT's proficiency in correcting mistakes and offering accurate definitions and examples for clinical studies. They concluded that NLP‐based models like ChatGPT hold significant promise for enhancing scientific literacy, exploring literature, and generating new research hypotheses. Shahsavar and Choudhury [6] stressed the need for policymakers and healthcare stakeholders to establish guidelines and ethical standards for using AI‐powered chatbots in healthcare. By refining ChatGPT to address specific medical inquiries, users can receive more reliable and valuable information for decision‐making processes. Bulck and Moons [48] assessed ChatGPT's responses to virtual patient prompts, finding them trustworthy and valuable. Notably, 40% of experts rated ChatGPT's responses as more beneficial than those from Google, highlighting its potential impact on patient care.
- **Improved medical education**: In five included studies, ChatGPT displayed notable effectiveness in enhancing various aspects of medical education. Huang et al. [9] reported that ChatGPT excelled in providing comprehensive drug counseling. The model accurately conveyed drug‐related information but did not consider patients' individual circumstances. Jamil S. Samaan et al. [28] noted that while ChatGPT often delivered accurate and reproducible responses, it occasionally provided incorrect information, indicating the necessity for healthcare providers to supervise its use. This supports ChatGPT's role as a supplementary tool rather than a replacement for professional medical advice. Both ChatGPT‐3.5 and ChatGPT‐4 demonstrated a robust understanding of core concepts in radiation oncology, as observed by Yixing Huang et al. [30]. Juhi et al. [45] found ChatGPT to be a somewhat effective tool for predicting and explaining drug‐drug interactions (DDIs), particularly beneficial for patients lacking immediate healthcare access. Cascella et al. [15] emphasized ChatGPT's capability to learn from its mistakes, effectively adjusting incorrect responses when prompted. Their study also indicated that ChatGPT could accurately discuss public health topics, offer precise definitions, and even provide examples of clinical studies. They concluded that NLP‐based models like ChatGPT hold significant promise for advancing scientific literacy, exploring literature, and generating new research hypotheses.
- **Improved accuracy**: In four included studies, ChatGPT demonstrated significant potential in improving diagnostic accuracy. For instance, Johnson and Goodman et al. [27] noted that the model maintained high accuracy across different question types and difficulty levels, with only a slight dip on more complex queries. Balas and Ing [49] emphasized the model's ability to learn from past cases, enhancing diagnostic precision in real time, especially for ophthalmic conditions. Rao et al. [41] highlighted ChatGPT's moderate accuracy in determining appropriate imaging steps for breast cancer screening and evaluating breast pain. Additionally, Amri and Hisan [44] observed that ChatGPT aids medical students by generating practice problems and benefits from the improved accuracy expected in GPT‐4.
- **Research assistance**: In four included studies [15, 26, 42, 44], ChatGPT demonstrated significant potential as a research assistant. It aids in selecting strong research ideas, writing concise research proposals, and composing the background sections for research projects. Additionally, it helps in formulating clear research objectives, constructing hypotheses, and suggesting various study designs and data analysis plans. ChatGPT effectively engages with higher‐order thinking questions and synthesizes complex data from electronic health records and clinical notes. Furthermore, it supports academic writing by refining texts, summarizing content, and generating abstracts.
- **Self‐learning**: In three included studies [15, 29, 48], ChatGPT demonstrated a remarkable capacity to learn from its errors and showed significant potential for improvement through the use of large language models and reinforcement learning from human feedback.
- **Summarization**: Two studies highlighted the summarization capability of ChatGPT. Cascella et al. [15] noted that ChatGPT excels in summarizing information into simple language, making it effective for communicating with patients and their families. Additionally, Amri and Hisan's [44] research demonstrated ChatGPT's promising potential in summarizing texts, particularly within the context of research.
- **Enhancement critical thinking**: Almazyad et al.'s [40] study demonstrated that incorporating ChatGPT‐4 into pediatric palliative care panel discussions could enhance critical thinking among medical professionals.
- **Interaction abilities**: Zuccon and Koopman [39] highlighted in their study that one of ChatGPT's standout features is its interactive capabilities. The model's ability to engage in multi‐turn conversations enables it to provide multiple pieces of evidence and clarify any unclear aspects of its responses.

### 3.4 Limitations of ChatGPT in Healthcare

In this section, the limitations of using ChatGPT in healthcare are mentioned. The most limitations reported in the studies included knowledge limitations and accuracy (*n* = 7), reliability (*n* = 7), and information sources and training data (*n* = 7). Following, the explanations related to each of the limitations are stated.

**Knowledge limitations and accuracy (*n*** = **14)** [15, 24, 25, 26, 28, 30, 32, 33, 34, 35, 37, 42, 46, 47]

- Limited knowledge in specific medical areas (e.g., gynecology, brachytherapy, dosimetry, clinical trials).
- ChatGPT content cannot be used as an alternative to the original reliable sources.
- Inaccurate content due to “hallucination” (believable but incorrect answers) and potential biases.
- Inability to determine uncertainty and its inconsistent accuracy.

**Reliability (*n*** = **12)** [6, 15, 23, 25, 26, 28, 31, 32, 35, 46, 48]

- Misleading responses due to slight variations in wording or tone.
- The source of information used by ChatGPT to produce responses is unknown, which may impact the reliability of its answers for certain topics.
- Dependence on user input and potential for unintentional inaccuracies.
- Cannot take responsibility for the content it generates.
- The model's inconsistency in accuracy poses a risk for decisions based on inaccurate answers.
- The risks associated with using ChatGPT in triage, clinical diagnosis, and patient care highlight reliability concerns.

**Information sources and training data (*n*** = **11)** [15, 23, 25, 29, 32, 38, 40, 44, 46, 47, 48]

- Lack of up‐to‐date information (based on GPT‐3 training up to 2021).
- Unknown sources of information used by ChatGPT.
- Can reproduce biases present in the data it was trained on.
- ChatGPT's errors may not be noticed by learners, affecting its use as an educational tool in primary care.
- Interpretability, reproducibility, and transparency are limited due to the lack of references in ChatGPT.

**Clinical decision‐making challenges (*n*** = **8)** [15, 25, 26, 29, 41, 43, 46, 47]

- Difficulty identifying futile care situations.
- Struggles with complex prescriptions and patient medication education.
- Cannot replace human doctors in primary care.
- Challenges in handling unpublished clinical contexts.
- Limited reliability for specific medical areas (e.g., cancer diagnosis).
- The model's frequent errors compromise its use as a decision support tool or educational assistant.
- The warning against using ChatGPT to explain medical concepts highlights the risks to clinical reasoning skills when relying solely on such systems.
- The presence of divergent opinions among experts regarding AI‐generated suggestions adds complexity to decision‐making.

**Education and monitoring (*n*** = **10)** [6, 24, 25, 26, 32, 33, 43, 44, 46, 47]

- Educating users about ChatGPT's limitations.
- Lack of awareness and education can hinder the acceptance of AI‐generated suggestions, emphasizing the need for ongoing monitoring and education in AI deployment.
- The output is based only on the libraries on which ChatGPT was trained.
- The responses are verbose and commonly generic.
- Risk of deterioration of critical thinking and communication skills among healthcare students.
- Cannot pass a national primary care examination.

**Responsibility and decision risk (*n*** = **8)** [6, 25, 32, 33, 40, 45, 46, 48]

- ChatGPT cannot take responsibility for content generation.
- Uncertainty in responses without an obvious way to determine model confidence.
- Difficulty in readability and understanding for the general population.
- The risk of misleading answers highlights the need for caution when using ChatGPT for health‐related queries.
- The risk of academic dishonesty involves decision‐making.
- ChatGPT's frequent errors compromise its effectiveness as an educational tool.
- Employing ChatGPT without empirical evidence may have unforeseen consequences.
- Overreliance on AI‐generated content could potentially lead future HCWs to reduced critical thinking and decision‐making abilities.

**Ethical and policy considerations (*n*** = **8)** [6, 15, 25, 33, 36, 38, 40, 44, 46]

- Data privacy risks.
- Need for guidelines and ethical standards.
- Legal concerns related to AI‐generated content.
- Plagiarism, copyright, and academic integrity.
- Early adoption of AI systems like ChatGPT without empirical evidence may have unforeseen consequences.
- The discrepancy between human perceptions of complexity and ChatGPT's performance at the subject level emphasizes the need for validation and careful application of NLP models in healthcare.

**Wrong reference list (*n*** = **4)** [25, 26, 31, 48]

- The references cited as evidence were either full of mistakes or completely fabricated.

**Patient safety (*n*** = **4)** [6, 25, 28, 46]

- Ensuring patient safety involves addressing ChatGPT's limitations, enhancing performance, and monitoring its impact.
- Ensuring patient safety involves addressing ChatGPT's limitations and promoting responsible use.
- Misinformation propagated by ChatGPT poses risks to patient safety.

## 4 Discussion

This scoping review has highlighted both the advantages and limitations of ChatGPT in the healthcare sector. The identified advantages were categorized into eight groups, while the limitations were divided into nine. The most commonly cited benefits were “CDS” and “improved medical education.” On the other hand, about half of the studies pointed out issues related to ChatGPT's “knowledge limitations and accuracy” and “Reliability” being the next most frequently mentioned limitation.

Our study demonstrated that ChatGPT holds promise for providing decision support across various health domains. In line with our study, Teixeira‐Marques [50] indicated that ChatGPT could assist otorhinolaryngology residents and specialists in making optimal patient decisions. Additionally, several studies [51, 52, 53] evaluated ChatGPT's effectiveness in supporting clinical decisions related to clinical vignettes, cardiology, radiology, and cancer reporting high accuracy. Bagde et al. [54] also highlighted ChatGPT's potential in medical and dental decision‐making, as well as its role in facilitating medical education and research.

Consistent with our findings, numerous systematic reviews have highlighted the potential applications of ChatGPT in medical education and health science research. Sallam [12] noted that ChatGPT benefits healthcare education by fostering critical thinking, enhancing personal learning, and supporting problem‐based learning. Xu et al. [55] demonstrated in their review that ChatGPT provides personalized learning support to medical students through its advanced natural language generation capabilities, enabling it to deliver accurate answers. It has shown significant utility in simulating clinical scenarios, facilitating teaching and learning processes. Zarei et al. [56] discussed the applications of ChatGPT in medical education, including improved interactions between medical students and patients, increased research opportunities, enhanced education quality, and personalized learning experiences.

In the study of Zarei et al., they also identified significant challenges in using this AI tool, such as limited access to reliable databases, ethical and transparency concerns, restricted information availability post‐2021, and the risk of generating AI hallucinations [56]. These findings align with our study's results. Additionally, our research demonstrated that ChatGPT has limited knowledge in certain specific medical fields, likely due to a lack of information in those areas. This indicates that this chatbot is most effective in domains where ample information is available. If the information is lacking, ChatGPT cannot provide assistance and is highly dependent on the data it has been trained on. Furthermore, ChatGPT is unable to assess uncertainty. Sharma [57] highlighted this inability to handle uncertainty as a limitation of ChatGPT, which can have detrimental consequences in healthcare settings.

This review identified several reliability issues, such as misleading responses due to slight variations in wording or tone, and the unknown sources of information used by ChatGPT. These factors affect the trustworthiness of its answers, particularly in critical areas like triage, clinical diagnosis, and patient care. The dependence on user input and the potential for unintentional inaccuracies underscore the need for cautious use of ChatGPT in healthcare. Despite its impressive performance, ChatGPT has been known to generate convincing yet erroneous information, which undermines its reliability, particularly in the healthcare sector [58]. Cong‐Lem et al. [59] identified five key limitations of ChatGPT in their review, with the most prevalent being accuracy and reliability. These concerns are particularly significant in critical domains like healthcare [60].

Our study has shown that one‐third of the studies aimed to assess ChatGPT's accuracy and reliability in responding to clinical queries from both patients and physicians. A study published in JAMA Network Open found that ChatGPT provided predominantly accurate information in response to diverse medical queries generated by physicians across 17 specialties [61].

In our scoping review, although the studies did not focus on a specific clinical field, the majority were conducted within the realm of medical research. Also, a review conducted by Rao et al. [62] highlighted ChatGPT's utility in analyzing large datasets, aiding in the development of novel research methodologies, and enhancing the efficiency of clinical trials. Another study conducted by Dave et al. [63] discussed how ChatGPT can assist in identifying potential research topics, supporting clinical and laboratory diagnosis, and providing updates on new developments in various medical fields. These applications underscore the transformative impact of ChatGPT on medical research, from improving data analysis to facilitating innovative research approaches.

### 4.1 Limitations

This review has several limitations. First, although it identified studies from four major databases, it is possible that some relevant studies were not included because they are not indexed in these databases. Second, the review does not evaluate the methodological quality of the included studies, limiting our ability to fully assess the generalizability of the findings. Future reviews should consider incorporating a quality assessment to address this gap. Third, the exclusion of non‐English records may have introduced selection bias, potentially affecting the comprehensiveness of the review. Lastly, we did not conduct thematic analysis. Additionally, we did not need PROSPERO registration since the study did not involve human subjects.

## 5 Conclusion

This scoping review has provided a comprehensive analysis of the advantages and limitations of ChatGPT in the healthcare sector. While the model shows significant promise in enhancing CDS and improving medical education, it is essential that its limitations in accuracy and reliability must be addressed. The findings underscore the need for careful integration of ChatGPT into healthcare practices, ensuring that its use is complemented by professional oversight and verification. As the landscape of healthcare continues to evolve, ongoing research and development will be crucial to harnessing the full potential of ChatGPT while addressing its inherent challenges. Future research should focus on improving the model's knowledge base, reducing hallucinations, and enhancing its ability to handle uncertainty. By addressing these challenges, ChatGPT could become a valuable tool in clinical decision support, medical education, and health science research.

## Author Contributions

**Seyyede Fateme Ghasemi:** methodology, conceptualization, writing—original draft, validation, data curation, software. **Parastoo Amiri:** methodology, conceptualization, writing—original draft, writing—review and editing, validation, data curation. **Zahra Galavi:** methodology, conceptualization, writing—original draft, writing—review and editing, project administration, supervision, data curation.

## Ethics Statement

This manuscript does not include any experimental work with animals or human subjects.

## Conflicts of Interest

The authors declare no conflicts of interest.

## Transparency Statement

The lead author Zahra Galavi affirms that this article is an honest, accurate, and transparent account of the study being reported; that no important aspects of the study have been omitted; and that any discrepancies from the study as planned (and, if relevant, registered) have been explained.

## Supporting information

Supporting Material 1.

## Data Availability

Data generated and analyzed during this study can be obtained from the corresponding author upon request.
