# Supplementary material for: Advantages and Limitations of ChatGPT in Healthcare: A Scoping Review
Source: Health Sci Rep. 2025 Sep 11;8(9):e71219. doi: 10.1002/hsr2.71219 (PMC12423551; doi:10.1002/hsr2.71219)
Supplement: Supplementary file 1 — Supporting Material 1. [file HSR2-8-e71219-s001.docx]

Search syntax in three databases

| Databases | Search syntax |
| --- | --- |
| PubMed | (“Chat gpt”[all] OR ChatGPT[all] OR GPT[all] OR "Chat Generative Pre-Trained Transformer"[all] OR Chatbot*[all] OR "medical chatbot"[all]) AND (Healthcare[all] OR "Health care"[all] OR Health[all] OR "medical education"[all] OR (Education[all] AND Medical[all]) OR "healthcare education"[all] OR "health care education"[all] OR "Clinical research"[all] OR Medicine[all] OR "public health"[all] OR (Health[all] AND Public[all]) OR Treatment[all] OR Prevention[all] OR Screening[all] OR "medical advice"[all] OR Triage*[all] OR "clinical workflow*"[all] OR diagnose*[all] OR self-management[all] OR "Self Management"[all]) AND 2020/01/01:2023/05/30[dp] |
| WOS | (ALL=(“Chat gpt”) OR ALL=(ChatGPT) OR ALL=(GPT) OR ALL=("Chat Generative Pre-Trained Transformer") OR ALL=(Chatbot*) OR ALL=("medical chatbot"))  AND  (ALL=(Healthcare) OR ALL=("Health care") OR ALL=(Health) OR ALL=("medical education") OR (ALL=(Education) AND ALL=(Medical)) OR ALL=("healthcare education") OR ALL=("health care education") OR ALL=("Clinical research") OR ALL=(Medicine) OR ALL=("public health") OR (ALL=(Health) AND ALL=(Public)) OR ALL=(Treatment) OR ALL=(Prevention) OR ALL=(Screening) OR ALL=("medical advice") OR ALL=(Triage*) OR ALL=("clinical workflow*") OR ALL=(diagnose*) OR ALL=(self-management) OR ALL=("Self Management")) AND PY= (2020-2023)) |
| Scopus | (TITLE-ABS(“Chat gpt”) OR TITLE-ABS(ChatGPT) OR TITLE-ABS(GPT) OR TITLE-ABS("Chat Generative Pre-Trained Transformer") OR TITLE-ABS(Chatbot*) OR TITLE-ABS("medical chatbot")) AND (TITLE-ABS(Healthcare) OR TITLE-ABS("Health care") OR TITLE-ABS(Health) OR TITLE-ABS("medical education") OR (TITLE-ABS(Education) AND TITLE-ABS(Medical)) OR TITLE-ABS("healthcare education") OR TITLE-ABS("health care education") OR TITLE-ABS("Clinical research") OR TITLE-ABS(Medicine) OR TITLE-ABS("public health") OR (TITLE-ABS(Health) AND TITLE-ABS(Public)) OR TITLE-ABS(Treatment) OR TITLE-ABS(Prevention) OR TITLE-ABS(Screening) OR TITLE-ABS("medical advice") OR TITLE-ABS(Triage*) OR TITLE-ABS("clinical workflow*") OR TITLE-ABS(diagnose*) OR TITLE-ABS(self-management) OR TITLE-ABS("Self Management")) AND (PUBYEAR > 2019 AND PUBYEAR < 2023) |
